# Design and development of a digital intervention for workplace stress and mental health (EMPOWER)

**DOI:** 10.1016/j.invent.2023.100689

**Published:** 2023-11-04

**Authors:** Christina M. van der Feltz-Cornelis, Jessie Shepherd, Jessie Gevaert, Karen Van Aerden, Christophe Vanroelen, Oriol Borrega Cepa, Laura González Recio, Renaldo M. Bernard, Ellen Vorstenbosch, Paula Cristóbal-Narváez, Mireia Felez-Nobrega, Carlota de Miquel, Dorota Merecz-Kot, Kaja Staszewska, Marjo Sinokki, Päivi Naumanen, Leona Hakkaart-van Roijen, Frédérique van Krugten, Marleen de Mul, Josep Maria Haro, Beatriz Olaya

**Affiliations:** a MHARG, Department of Health Sciences, Hull York Medical School, University of York, York, United Kingdom; b Interface Demography, Vrije Universiteit Brussel, Brussels, Belgium; c Òmada Interactiva, SLL, Barcelona, Spain; d Swiss Paraplegic Research, Nottwil, Switzerland; e Research, Innovation and Teaching Unit, Parc Sanitari Sant Joan de Déu, CIBERSAM, Sant Boi de Llobregat, Spain; f Institute of Psychology, University of Lodz, Lodz, Poland; g Nofer Institute of Occupational Medicine, Lodz, Poland; h Turku Centre for Occupational Health, University of Turku, Turku, Finland; i Erasmus School of Health Policy and Management (ESHPM), Erasmus University Rotterdam, Rotterdam, the Netherlands

**Keywords:** Digital intervention, Workplace, Work stress, Mental health, Wellbeing, Qualitative research, Employees

## Abstract

**Purpose:**

We describe the design and development of the European Platform to Promote health and wellbeing in the workplace (EMPOWER) digital intervention that provides an integrative user programme meeting the needs of employees and employers in addressing work stress.

**Results:**

A user-centred design process was followed from January 2020 until November 2021. A tailored algorithm was developed to provide support at the individual employee level and the company level. Each element of the digital intervention was developed in English and then translated in Spanish, English, Polish and Finnish. The digital intervention consists of a website and a mobile application (app) that provides algorithm-based personalised content after assessing a user's somatic and psychological symptoms, work functioning, and psychosocial risk factors for work stress. It has a public section and an employer portal that provides recommendations to reduce psychosocial risks in their company based upon clustered input from employees. Usability testing was conducted and showed high ease of use and completion of tasks by participants.

**Conclusion:**

The EMPOWER digital intervention is a tailored multimodal intervention addressing wellbeing, work stress, mental and physical health problems, and work productivity. This will be used in a planned RCT in four countries to evaluate its effectiveness.

## Highlights

• Digital health applications for the work setting often don't use validated materials.
• The EMPOWER app advises individual employees and employers on workstress.
• It was developed in English and translated in Spanish, Polish, and Finnish.
• It aims to improve workstress and mental wellbeing in the workplace.
• It will be evaluated in an RCT in the United Kingdom, Spain, Finland and Poland.

## 1 Introduction

Depression and anxiety have an enormous impact on the well-being of employees, their employers, and society (GBD 2019 Diseases and Injuries Collaborators, 2020). Symptoms of depression and anxiety are related to work stress and lowered productivity while at work, which may have a substantial economic impact (Adler et al., 2006; Wang et al., 2023; Lerner et al., 2010; Henderson et al., 2011; Harvey et al., 2013). For example, 51 % of work-related ill health and 55 % of lost workdays reported in the United Kingdom in 2019 were due to anxiety or depression, both of which can be treated successfully and might have been prevented (Health and Safety Executive, 2020).

Addressing work stress and mental well-being at the workplace might improve work functioning. Digital interventions have been introduced with that objective and small to medium size effects have been reported (Stratton et al., 2021; Heber et al., 2017; Miguel et al., 2023). They tend to have high attrition rates, for example, 48.5 % of participants were reported to have dropped out of one intervention (European Commission, 2018). Although some digital interventions promise to be effective in addressing work stress or mental disorders at the workplace, there is a general lack of interventions that address both symptoms in employees and psychosocial risk factors at the workplace (Torous et al., 2020). Also, they generally focus on employees and do not support employers in improving well-being in their workforce (Volker et al., 2015). Additionally employers report a lack of knowledge and ability to address work stress and well-being (Deloitte Global Human Capital Trends, 2017). Moreover, there is a call for research to understand which factors contribute to the variation in effectiveness of particular interventions depending on the mental health area and characteristics of participants and interventions, in other words, for providing modules based on triage (Phillips et al., 2019).

A digital interventions' ability to be adapted to the diverse contexts in which they are employed is fundamental to practical application. When using an intervention cross-culturally, the translation of language is a complex process, since there are differences in the connotations of words, particularly for mental health and wellbeing (Beck et al., 2003). It is important then to not translate the words alone, without consideration of cultural context, as this can lead to misunderstanding or for the intervention to not function in the way intended (Resnicow et al., 2000).

To cover a range for our cultural validation, we also aimed to allow for differences in legislation regarding sickness absence, which varies greatly from country to country. Some countries have generous wages paid during illness. For example, the Netherlands, where in the event of illness, wages are paid for 2 years, from the 2nd year generally at 70 % of the salary; and Germany, where wages are paid for 6 weeks to a maximum of 78 weeks in 3 years, for 70 % of the salary. On the other hand, in a country like Sweden employers pay wages for 14 working days at 80 %, but some employers don't; after that the employee can ask for benefits from the Swedish Social Insurance Agency (European Commission, 2023a).

Four countries were chosen to participate in the development and cultural validation of the digital intervention: the United Kingdom (Anglo-Saxon model), Spain (Mediterranean model), Finland (Nordic model) and Poland (Central-Eastern model). Their regulations for sick leave are as follows:

• The UK, which has 28 weeks of continued wages payment with max. £109.40 per week gross, independent of actual salary, in case of illness (Statutory Sick Pay) (Gov.uk. Working, Jobs and Pensions, 2023).
• Spain, where the first 15 days of illness are paid. Full salary is covered via the country's social security system. It is standard practice for employers to follow this mandatory minimum entitlement (European Commission, 2023b).
• Finland, where wages are paid for 10 working days at 100 % of the salary. Most employers also pay the full salary during the first one to two months. A doctor's certificate is required for the period during which you are unable to work. Social security will pay sickness allowance for a maximum of one year (300 working days). If you receive pay during the period of illness, social security will pay the compensation to the employer. To receive partial sickness allowance, working hours and pay must have been reduced by 40–60 %. Partial sickness allowance can be claimed for a maximum of 120 working days (European Commission, 2023c).
• Poland, where Employers are responsible for paying employees on sick leave 80 % of their salary for the first 33 days of illness in a calendar year, regardless of breaks. The amount increases to 100 % if the employee becomes ill during pregnancy or if their illness were related to accidents at work or commuting to or returning from work. The difference, in this case, is covered by the Social Security Institute. After the first 33 days, the Social Insurance Institution covers the leave, generally at 80 % of the base pay for a maximum of 182 consecutive days per year (inclusive of the 33 days paid by the employer). For employees aged 50+, the company must only cover the first 14 days of sick leave. The rest is covered by Social Security (Employee leave Entitlement, 2023).

The European Intervention to Promote Wellbeing and Health in the Workplace (EMPOWER) intervention (Olaya et al., 2021) aims to address work stress and well-being for employees at the individual level and to explore opportunities for employers to identify and address psychosocial risk factors in the workplace that might impact well-being. The intervention was designed to be implemented in small and medium enterprises (SMEs), which are organizations with fewer than 250 employees and an annual turnover not exceeding EUR 50 million (European Commission, 2020b) and public agencies, which are organizations with a public legal personality dependent on the administration for the performance of activities within the competence of the region or country under a functional decentralization regime. Large companies (i.e. ≥250 employees) can also participate despite not being the main focus. This will be done in the United Kingdom (UK), Spain, Finland and Poland, that represents diverse welfare and health service models.

### 1.1 Study aim

In this study, we aim to describe in detail the design and development of the digital intervention in English, and translate and culturally validate each component of the intervention in Spanish, Polish and Finnish.

## 2 Materials and methods

We aimed to develop a new digital intervention, which included a website and a mobile app. The design took one year and three months (Jan 2020 - March 2021). We followed a mixed method, co-design approach. We based the intervention on several building blocks:

(1) the outcomes of a systematic review (Byrne et al., 2022), that defined tailored, personalized digital interventions;
(2) material from a study improving work productivity in common mental disorders in the workplace with a digital intervention (Torous et al., 2020), and
(3) publicly available intervention materials adapted as needed in English.

In our design of the digital intervention, we applied personalization and tailoring as follows: 1) an algorithm to triage based upon assessment or questionnaire scores and to provide summarised feedback, 2) participant choice for part of the modules; 3) automated messages to users, 4) indirect support by the managers or employers, based on recommendations from the app, to deal with psychosocial risks detected in that particular company.

The digital intervention was developed in the following eight months (April 2021–November 2021) after translation and cultural adaptation of the intervention for the other three countries planned to participate in the subsequent RCT to evaluate the effect of this intervention in small and medium companies, public agencies and large companies (Olaya et al., 2021). We followed the APA approach for reporting of the qualitative methods and results (American Psychological Association, 2020).

### 2.1 Design of the intervention

**Fig. 1:**
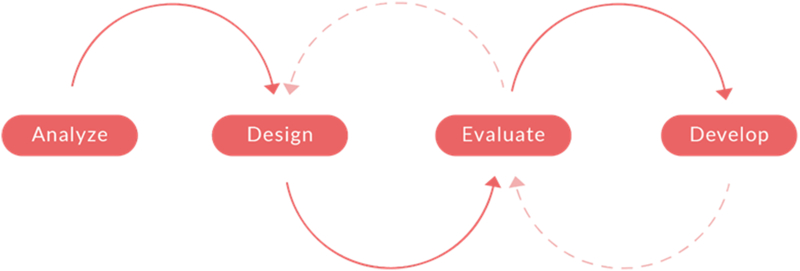
Four-step user-centered design process.

A four-step user-centered design process was followed to create designs for the digital intervention's website and app (Abras et al., 2004). This process is illustrated in Fig. 1 and focuses on closely matching end-users needs and context of usage to the design (Biron et al., 2004).

Designers first analysed the needs, goals and context of usage for target users (workers) and key stakeholders (employers and managers). This general requirement gathering exercise for the intervention involved a multidisciplinary team consisting of researchers, developers and clinicians (occupational physicians and psychiatrists). The app's design was intended to meet the needs of persons with different digital literacy levels and various job and demographic profiles. User-experience requirements were mainly persona-based (Pruitt and Adlin, 2010) and relied on user-interface design principles (Nielsen et al., 2019). Hence, the planning process was adapted to accommodate the national shutdown and necessary safety restrictions. We created several target-user profiles based on assumptions about users, results from published literature on mental health apps, and past reports of user experience with these apps. These profiles were designed to represent the needs of prototypical target users of the app and helped us build a beta version of the digital intervention, which we tested during the usability testing. Several high-fidelity functional prototypes were then designed and developed as a non-interactive web-based prototype and then underwent usability testing by end-users and an expert evaluation by key stakeholders, such as Human Resources managers, unions representatives, and health professionals i.e. occupational physicians and psychologists.

## 3 Theory

### 3.1 Translation

The EMPOWER intervention will be evaluated in an RCT in four countries representing different European welfare and health service models (Olaya et al., 2021): Finland (protective and universal regimes in terms of welfare provision), Spain (a fragmented system of welfare provision, and strong reliance on family and charitable sector), Poland (an underdeveloped welfare system but strong labour market institutions and solid industrial economy), and the United Kingdom (National Health Service, short-term benefits for sick-listed employees) (Alvarez-Galvez et al., 2014). Hence, due to expected cultural differences between these four countries, we followed a multistep or stage model framework (Castro et al., 2010) inspired by both the “cultural sensitivity approach” and “negotiated consensus” approach (McGreevy et al., 2014). There is plenty of evidence showing these methodologies to be commonly applied in the process of cultural adaptation (Beck et al., 2003; Castro et al., 2010; McGreevy et al., 2014; Spanhel et al., 2021; Escoffery et al., 2019; Barrera and Castro, 2006). Together, they involve four steps:

1) forward translation (the process in which a text (e.g., in English) is translated to the native language of the translator) with cultural adaptation to make content more understandable for the participants from that setting (Phillips et al., 2019), following a cultural adaptation protocol that was developed for this purpose;
2) negotiated consensus: discussion between research teams on challenging concepts and documentation of such modifications to maintain the core parts of the materials as well as allow for cultural sensitivity (McGreevy et al., 2014);
3) pre-testing: evaluation by potential end-users and experts through online focus groups or functional alternatives (e.g., interviews, written consultation) in all countries. Seeking input from key actors (i.e., potential end-users, employer representatives and legal experts), can help inform and further tailor the intervention, which is known to increase positive results after the intervention (Sorensen et al., 2016).
4) modification of the material based on end-user feedback (El Masri et al., 2019). This phased approach allowed for linguistic translation and cultural adjustment while maintaining the empirical and institutional source material.

For this approach, we used a Translation and Verification Follow-up Form based on the European Social Survey Translation Process (Mohler et al., 2016), which was co-developed with an external service provider specialized in language solutions for linguistic and cultural comparability (Mohler et al., 2016). This ensures continuous documentation on the types of modifications that have been made to the base version of the intervention and records why modifications have been made. Systematically documenting the steps taken to ensure cultural sensitivity is a common recommendation in literature on cross-cultural research practices (Mohler et al., 2016; Escoffery et al., 2018).

This verification form is a way of establishing validity for our cultural sensitivity approach. It ensures replicability and reproducability by tracking any potential modification that was made to the intervention as a result of cultural sensitivity.

This cultural sensitivity approach allowed for the adaptation of both surface structure (e.g., adaptations of language, names, activities etc.) and deep structure adaptations (e.g., adaptations in specific cultural health behaviours and patterns) consistent with (Beck et al., 2003) while continuously considering the ‘fidelity-adaptation dilemma’ (Castro et al., 2010) by deciding when to adapt specific components and when to stay true to the core elements of the intervention.

Finally, experts' evaluation of the material was arranged by organising online consultations with relevant local stakeholders in each of the four countries. The main goal was to receive feedback on the EMPOWER intervention and the content of the intervention materials from stakeholders with different areas of expertise to improve the intervention before its actual implementation. The final procedure for organising the local stakeholder consultations was composed of six steps (preparation, recruitment, first virtual meeting, execution, second virtual meeting, and reporting). It was developed with the specific aim of ensuring a high degree of uniformity between the four settings. In the first phase, each of the country teams selected, based on a set of standard profile requirements, 6 to 10 potential participants to be included in the consultation round for their country. These individuals (at least: one academic from outside the research team, one employee representative, one employer representative and one occupational health expert or occupational physician) were invited to participate in the consultation group using a standard e-mail explaining the goal and the procedure. The four country teams then organised a first virtual meeting with all members of their local consultation group, in which parts of the material of the EMPOWER intervention were presented to the participants. These virtual meetings were based on a standard script and identical materials to ensure consistency between the four countries. The meetings were also recorded to enable accurate processing of the information provided by the participants later. In the execution phase, the participants of the stakeholder consultation groups received an e-mail containing the materials selected for revision and a standard set of questions about these materials.

Literal answers were collected and summarised afterwards by each research team to be presented and discussed in the second and final virtual meeting. This second meeting allowed for a group discussion about the EMPOWER intervention and the materials that were reviewed. The results of the local stakeholder consultations were then summarised in a comprehensive report, with specific attention to the remarks made by the local stakeholders and the corresponding changes made to the materials.

During the EMPOWER digital intervention creation, it was essential to consider gender and cultural differences that could impact successful implementation. During the development of the intervention, cultural appropriateness and institutional context were considered for each of the four cultural settings (Spain, Finland, United Kingdom, and Poland). The intervention was carefully adapted to ensure the usability, acceptability, and adherence for each setting and comparability of the material across all four settings.

The considered adaptations included ways of rephrasing parts of the source materials or adding definitions and synonyms to complex concepts to ensure comprehension among the target population. Other factors of usability, like the structure of the text, which was deemed overly schematic by potential end-users, and overall framing, which end users felt was too ‘negative’ (i.e., focus on ‘illness’ rather than ‘wellness’), were discussed and adapted. Unique practices and ‘social codes of conduct’ within each setting were also considered (e.g., in the Spanish text, the mention of standing desks was limited because it was not a common practice in Spain). Further adaptations were related to the occupational health and safety regulations for each of the four settings (e.g., the equality policy and occupational health care were elaborated on in Finnish documents, and specific employee break regulations were clarified in Polish documents). In addition, cultural adaptation considered inherent characteristics of the organisational culture and worker characteristics, particularly gender.

### 3.2 Usability testing

Qualitative usability testing was performed on a website-based non-interactive prototype and subsequently on a software-based interactive beta version of the EMPOWER app. The usability testing aimed to assess both in the workplace to report on user experience and to gather recommendations from the participants. The specific aims of each phase of the usability testing and variables explored (Zhang and Adipat, 2005) are mentioned in Table 1.

**Table 1:** Aims of prototype and beta testing.

	Usability attributes	Definition of attributes
Prototype testing aims	Branding used in the app	Look of pictures, text, and layout
	Comprehensibility (in English only)	Users understanding of the content in a mobile format
	User satisfaction with material	Users attitude when using an app
Beta testing aims	Comprehensibility (in all four languages)	Users understanding of the content in a mobile format
	User satisfaction with material	Users attitude when using an app
	Simplicity	The ease with which users complete assigned tasks
	Effectiveness	The extent to which users are able to complete goals in the app

The English-only prototype testing was completed by sending participants a link to the prototype and a survey asking questions regarding the branding, comprehensibility, user satisfaction and recommendations for improvement. Survey responses were analysed and then incorporated into the design of the beta version of the app.

The usability testing for the beta version of the EMPOWER digital app took place in Spain, the United Kingdom, Finland, and Poland. Participants aged 18 or over were invited. A minimum of five to ten participants per site was aimed at as is common in usability testing (Six and Macefield, 2016). Participants were advised that all answers given in the survey were anonymous and that no personally identifiable data was collected. Volunteers were invited to access and use parts of the app's beta version in three iterations and a survey that guided them through the testing tasks. The survey was delivered by Google form. Survey responses were analysed to determine comprehensibility, user satisfaction, simplicity, and effectiveness as defined in Table 1, including ease of use, task completion, and usefulness of material in the app. Users were also asked open-ended questions around their recommendations for improvements.

## 4 Results

**Fig. 2:**
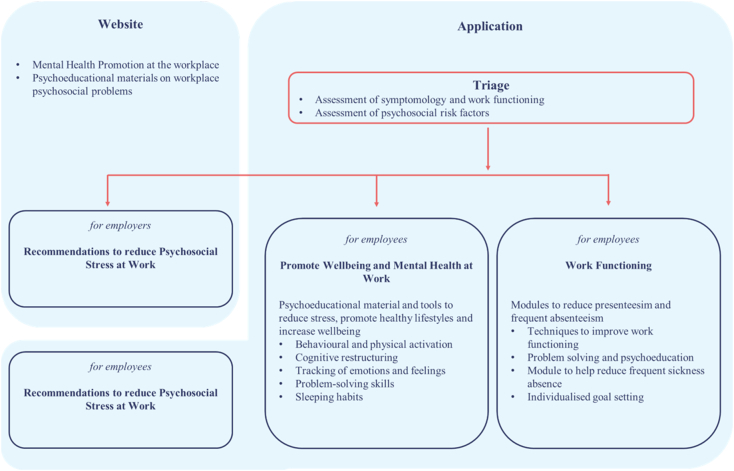
EMPOWER digital intervention.

The design and development process yielded the following EMPOWER intervention, as shown in Fig. 2. That provides an overview of the components of the digital intervention and how each level connects to the others.

### 4.1 EMPOWER digital intervention

The EMPOWER digital intervention has a website and app that are linked by assessment and triage. The main website is public and not individualised, providing a digital mental health awareness campaign at the company level that supports employees and employers in addressing work-related stress and mental health issues.

An employer portal within the website is designed to provide individualisation for employers without impacting employee confidentiality. It includes a brief assessment of psychosocial risk factors in the workplace by the employees, complemented by tailored recommendations for employers. It provides summarised feedback based upon their employees' assessment of psychosocial risk factors. This feedback is provided without identifying information and gathered using all participating employee responses to protect employees' confidentiality. This level is only accessible to employers who will participate in the planned EMPOWER RCT.

The app portion of the EMPOWER intervention is available for use by individual employees in participating companies. An algorithm was developed by the research group that uses outcomes from triage assessment tools to individually tailor content modules to each employee based on symptomatology and work functioning. Each user, therefore, has an individualized experience to match symptomatology and need.

### 4.2 Components

The intervention combines four components, that are described in more detail below.

1. A component of mental health promotion at the workplace that includes raising awareness about workplace mental health
2. A component to screen for psychosocial working conditions and provide recommendations to address those for employers and employees
3. A component to screen for mental health, physical health, and absenteeism of employees and provide recommendations to promote well-being and mental health in employees
4. A work functioning component to help deal with work-related problems such as presenteeism and frequent absenteeism

#### 4.2.1 Mental health promotion and raising awareness at the workplace (MHPA)

Psychoeducational materials were explicitly designed to increase awareness about mental health in the workplace, including the following sections, which provide information, examples, and advice: *What is a healthy workplace*?; *What is good mental health*?; *Why mental health matters*; *Workplace bullying*; *Types of mental health conditions*; *Workplace stress*, “*are they ok*?”; *Starting a conversation*; *Helping a workmate*; *Legal rights and responsibilities*. This material is offered on the public website.

#### 4.2.2 Screening instrument and recommendations for psychosocial working conditions (PSWS)

To raise awareness for factors that lead to mental ill-health at work, psychosocial working conditions and how they shaped the health and well-being of employees were explored in material publicly available on the website. This was meant to familiarize both employees and employers with psychosocial risk factors which may appear at work and their consequences for work functioning and health. These educational materials included the following topics: work overload, role ambiguity, conflicts at work, social support, professional development, and promotion (career issues), presenteeism, atypical working hours, remote work, burnout, sexual harassment, bullying, aggression, and workplace traumas.

**Fig. 3:**
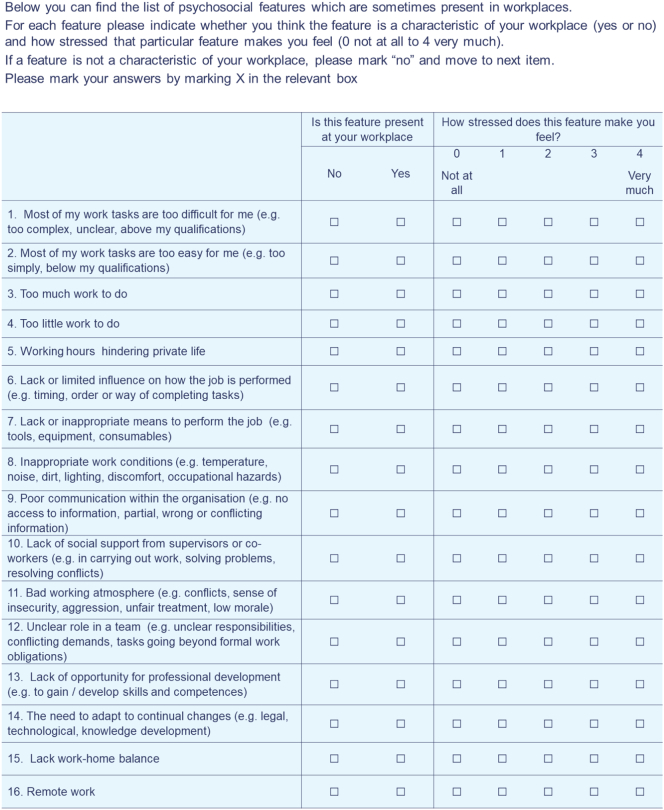
Psychosocial stressors at work scale.

We proposed a module devoted to screening universal psychosocial working conditions, which were thought to be potentially stressful. This proposal was conceptually grounded in the typology of workplace stressors developed by Cox (Cox et al., 2003) and The European Framework for Psychosocial Risks Management PRIMA-EF (Leka et al., 2008) and supported by a large body of research which showed direct relationships between workplace stress and burnout, depression, anxiety, adjustment disorders, somatisation, chronic fatigue, psychotropic drugs consumption and many other conditions (Godin et al., 2005; LaMontagne et al., 2010). The resulting module consisted of a Psychosocial Stressors at Work Scale (PSWS), individual recommendations for employees and employers based on the screening results and supporting educational materials for the website. The PSWS was developed entirely for the purpose of the EMPOWER project. This was done because existing tools measuring the burden of stress at work are either short but very generic which makes it impossible to design an intervention or very detailed and long, which are over burdensome on users. It is based on the Subjective Work Characteristics Questionnaire (SWCQ) (Dudek et al., 2004) and Psychosocial Risk Scale (PRS) (Mościcka-Teske, 2014) tools for the assessment of psychosocial stress at work. The Psychosocial Stressors at Work Scale consists of 16 items selected by six experts in occupational and health psychology from the original SWCQ and PRS. The psychosocial stress measure should, on one hand, give enough information for designing sound intervention at the workplace aimed to elimination or reduction of stress factors related to work and on the other hand be short enough not to burden the users of Empower Platform. The used version of the PSWS is shown in Fig. 3. The chosen items describe universal stressors which may appear in any job. The development was guided by three basic assumptions:

(1) the screening tool should be universal, i.e. it can be used in any workplace;
(2) it should be short so as not to burden employees,
(3) screening results should be easily translated into recommendations enabling changes to be introduced at the individual and organisational levels.

**Fig. 4:**
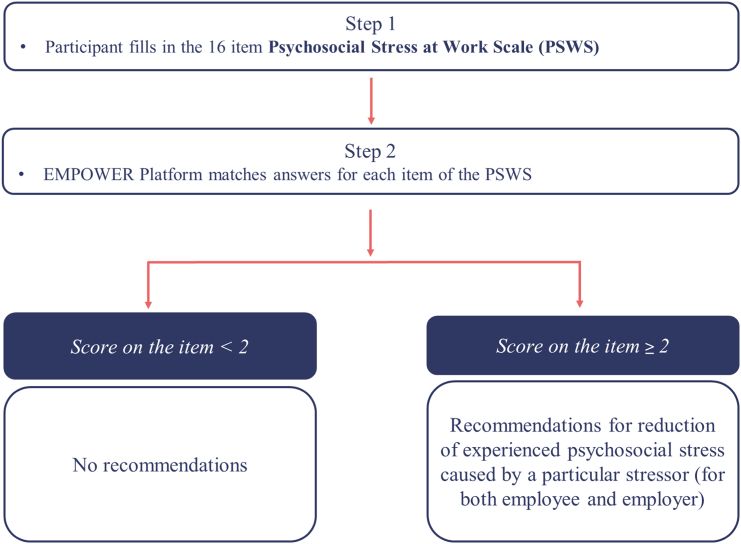
Algorithm for feedback based on the total score of Mini-PSWS.

The general score of the PSWS is calculated. Then, matching the testing results with recommendations for employees is based on the threshold of ≥2 points. So, if an employee states that their level of stress is ≥2 points on one answer scale, they will receive recommendations related to that particular problem at work, as presented in Fig. 4.

**Fig. 5:**
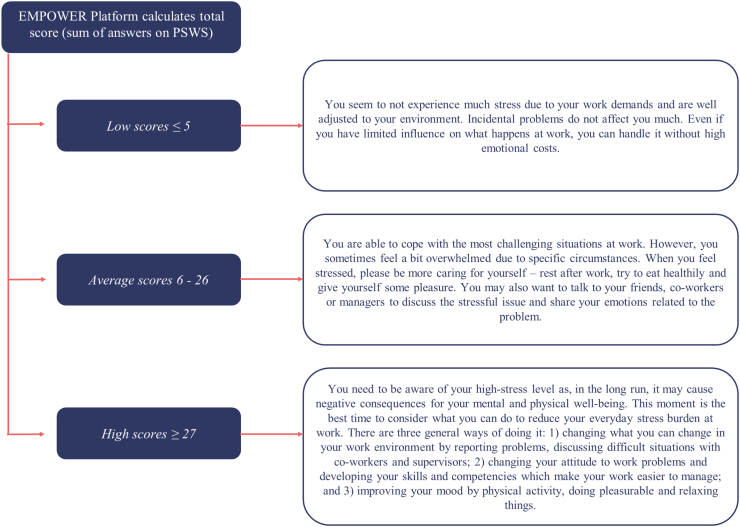
Example of recommendations based on total score.

Then, based upon sum scores for all questions taken together, general recommendations would be provided as shown in Fig. 5.

The PSWS questionnaire for employees is delivered through the app, and they also receive the tailored recommendations via the app.

Through a separate, dedicated and secure part of the EMPOWER website, employers will receive general information about the five most common stressors regarding occupational stress in their company after the period in which their employees could fill in the PSWS. This information will also be available for employees to ensure a transparent flow of information. Employers will receive recommendations based on the results of their employees.

Each recommendation describes the actions that can be taken to improve working conditions in the company. The system will not provide information on sources of stress if the number of employees is less than 10 to protect the anonymity of workers. In such cases, employers will receive a more general summary of what can be done to reduce the stress burden at work. Additionally, employers will be provided with a diagram on different steps that they can take to implement the EMPOWER intervention. They will also be provided with country-specific institutions or consultants that could help the companies with this. This way, employers were supported to fulfil their legal duties in relation to national law to address health, mental health and safety at work. This can especially be helpful for SMEs, which generally do not have the resources to make use of the offer of specialists providing stress prevention programmes in the workplace.

**Fig. 6:**
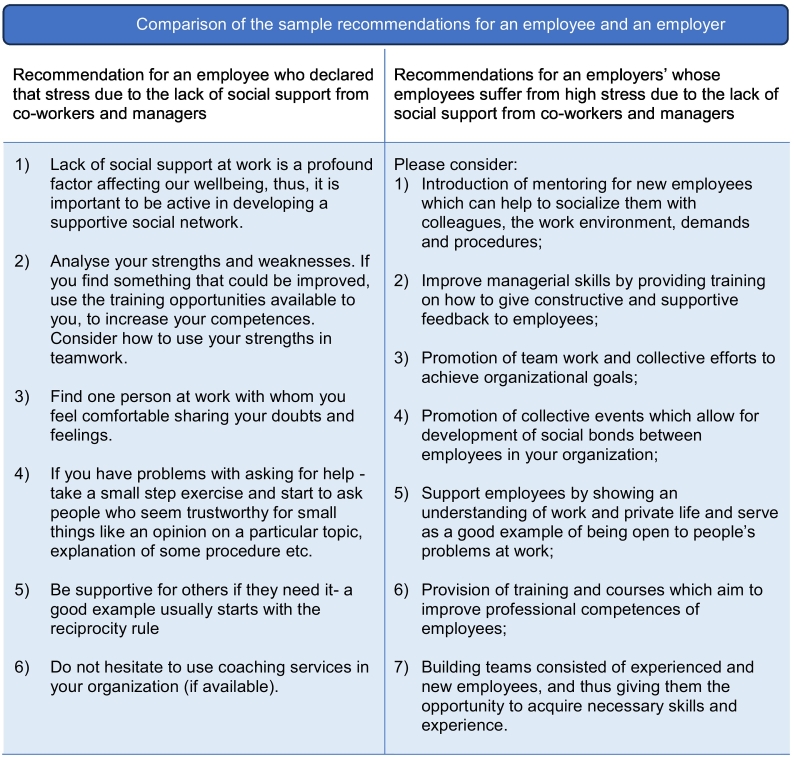
Comparison of the sample recommendations for an employee and an employer.

Examples of recommendations for the employee and employer are presented in Fig. 6.

All recommendations were the result of consultation with experts (occupational health professionals, researchers, representatives of employees and employers) in Poland, United Kingdom, Spain and Finland.

#### 4.2.3 Promoting well-being and mental health at work (PWMH)

Promoting Well-being and Mental Health (PWMH) is the third component of the intervention. This intervention is directed toward Small-to-Medium Enterprises (SMEs) with up to 50 (small) or up to 250 (medium) employees (European Commission, 2020a), and public agencies; it has an individual approach and was designed based on strategies and techniques of the Cognitive Behavioural Therapy (CBT) model. Previous evidence suggests that digital interventions that include a variety of mental health interventions, rather than a single mental health intervention, have a higher success rate (El Masri et al., 2019) and a higher participation and adherence rate (Robroek et al., 2009). Additionally, health promotion is more effective if it provides individuals with an array of tools, and the individual is thereby empowered to use the more appropriate approach for their situation (El Masri et al., 2019).

PWMH presents several blocks of psychoeducational and practical contents (e.g., relaxation exercises, breathing techniques), allows for tracking of daily-life moods, promotes healthy sleeping habits, and encourages the development of personal attitudes and skills (e.g., problem-solving strategies, cognitive restructuring techniques) in order to manage symptoms of stress, anxiety and depression. All contents of this intervention were designed and presented using gamification elements to promote self-learning and ongoing use and adherence to the program. The psychoeducation material of this module was created to enhance literacy, attitudes and supportive behaviours about well-being and mental health and includes information on core constructs of the intervention (i.e., stress, depression, anxiety and insomnia). The content offers a non-expert definition of each construct, information regarding signs, symptoms, potential causes and practical strategies.

#### 4.2.4 Work functioning (WF)

The Work Functioning (WF) component is the fourth intervention component. It was designed to provide participants with targeted prevention strategies and to support employees with presenteeism or frequent absences from work. This intervention component is tailored to the individual employee's needs based upon assessing their mental and physical health, presenteeism and absenteeism when they begin using the app. The intervention builds on interventions designed to address the most common mental health symptoms, such as stress, anxiety, insomnia, and depression, that were previously tested and proven effective and cost-effective in improving well-being and return to work in sick-listed employees with mental disorders (Torous et al., 2020; Robroek et al., 2009). This component also combined interventions to address the most common comorbid conditions based upon triage (Torous et al., 2020; Lokman et al., 2017). It encouraged employees to seek guidance and the help of health professionals when appropriate and was designed to complement treatment provided by a health professional.

The Work Functioning (WF) element of the app incorporated education about work functioning in case of stress at the workplace and how to deal with mental health problems in the workplace in interactive, tailored modules designed to correspond to participants' symptomatology.

The goal-setting section was designed to last three to six sessions. It involved four main elements:

1) Describing the problem,
2) Creating a goal to focus on,
3) Creating an action plan,
4) Self-evaluation of steps taken.

Each element contained advice and encouragement to assist the participant in skills development and building self-awareness.

Participants were allocated goal sessions during the initial planning for the WF goals setting based upon initial risk assessment scores. The goal-setting feature was designed to be sequentially provided after another module was completed. This initial plan was amended following feedback from the development team. WF goal setting was then changed to appear as a general tool in the app and provided concurrently with other materials. This update allows all participants to use the goal setting and repeat the goal setting if they decide to work on more than one goal or feel they need to re-evaluate their initial choice, enabling them to self-determine how to use the app according to their needs and desires. It provides access to this valuable tool to all participants rather than a subset of participants. This change in design is an example of how the needs and feedback from the target population guided the development teams' decision-making.

### 4.3 Triage design

A triage process was designed to guide the employees through the different modules of the app. App content was put together based upon initial user assessment and allocation of material is made through the use of an algorithm. The triage is based on the initial user assessment which involves questions on work functioning, physical and mental health, and comorbidity. Three core categories were created; within them were multiple user profiles allocated based upon assessment scores. It should be noted that while the app was designed to meet individualised user needs, it was not designed to meet the needs of those with suicidal ideation or suicidal intent. If a user selected that they experience suicidal ideation or intent when filling out assessment questionnaires, they received a popup message encouraging them to speak with a medical professional who can provide treatment and monitor them and informing them that although the EMPOWER app was not designed to address their specific needs, they were allowed to continue to use the app if they choose to.

The three core categories for users are:

A) participants who identified no current issues that affect their ability to get work done at their normal level in the 30 days prior to the assessment;
B) participants who identified they have experienced a problem that led to difficulty completing as much work as normal in the 30 days prior to the assessment; and
C) participants who identified they missed work more than three times in the last 12 months as a result of being sick, may or may not have also identified as having a problem that has led to difficulty completing as much work as they normally do in the 30 days prior to the assessment.

**Fig. 7:**
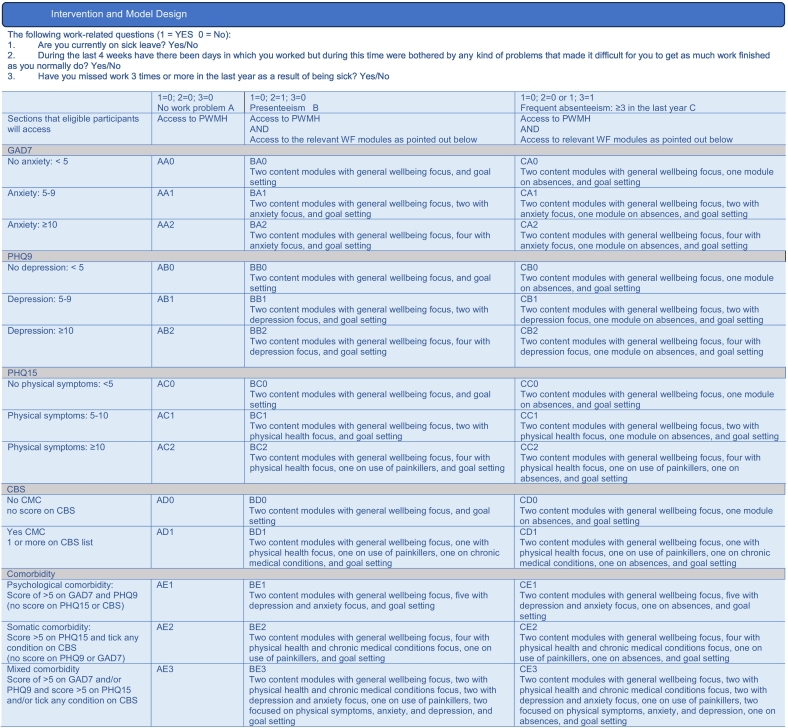
Intervention and Model Design.

The period of 15 days or less of absence from work is considered appropriate for an early intervention, designed to prevent longer-term sickness absence (Lokman et al., 2017). Within each of the three main categories, there were multiple user profiles which were allocated to the participants based on their reported symptoms and assessment scores. The content offered within each profile was tailored to the participants' needs and the intensity of symptoms reported. Triage of work functioning and symptomology was based upon users' response to validated questionnaires (GAD7, PHQ9, PHQ15 (Kroenke et al., 2010), Checklist chronic disease (CBS, 2005) exploring physical and mental health and work functioning as mentioned in Fig. 7.

The material provided within the intervention was chosen based on triage using participant initial assessment scores. The higher the participant assessment scores, and the more symptoms are reported, the more tools and psychoeducation were offered. At the first intervention level, participants will have access to psychoeducational materials and tools related to their symptomatology and needs. In addition, if a participant's risk assessment showed comorbid conditions, there were specific modules designed for combinations of symptoms. The design of the app, allowed for a tailored user experience, which has been shown in past literature to improve mental health, increase wellbeing, and reduce frequent absences (Volker et al., 2013).

As examples of this triaging, in the app a participant whose assessment outcome suggested they had minimal symptoms of anxiety or depression received material about continuing to access appropriate supports, increasing their resilience to potentially prevent maladaptive symptomology developing, and interactive goal setting. A user whose initial assessment outcome suggested high levels of anxiety, depression, or comorbidity, for example, was provided with psychoeducation, skills-building modules, examples of symptomatology and ways to combat it, and interactive goal setting. This variation in intensity of support as well as material which connects with participant symptoms, was an innovative and potentially highly effective way to design a workplace intervention to target mental health and absences.

### 4.4 Differentiation within the app based upon target group profiles related to digital literacy

**Fig. 8:**
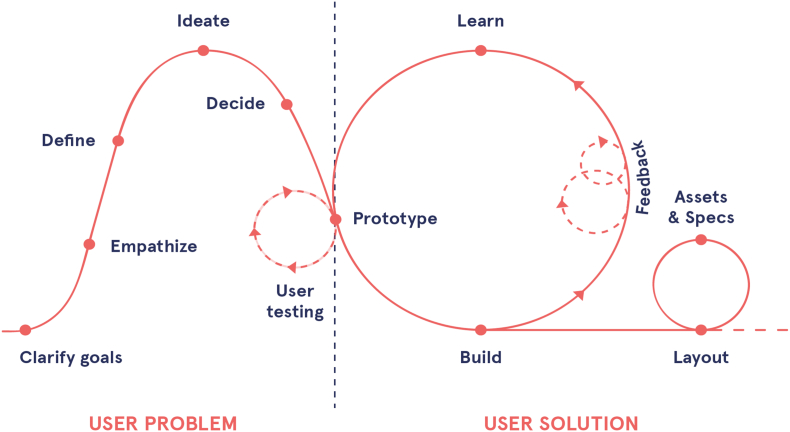
Agile design process as used for the app.

Initially, we created a user journey to represent a general profile of a potential EMPOWER intervention participant in order to estimate the general needs of users. Fig. 8 is an illustration of the design process used when creating the app (Vargas-Prada et al., 2016). During the initial planning phase, we created a first version of the navigation flow based on the content and benchmarking (a comparison) of similar pre-existing apps (i.e., Return to Work (Torous et al., 2020), Headspace (HEADSPACE, 2021), Calm (Huberty et al., 2019), or Downdog (Downdog, 2021). We then created the first wireframes (initial illustration of the screens and navigation) of the EMPOWER app, allowing us to investigate potential issues, such as bottlenecks (when the developed app does not meet the needs of the stakeholders). We followed the method of agile manufacturing for this as indicated in Fig. 8 (Gunasekaran, 1999).

A total of 25 stakeholders participated in the local consultation groups (9 in Poland, 4 men and 5 women; 8 in Finland, with 3 men and 5 women; and 8 in Spain, 4 men and 4 women). They were from the following fields: occupational health experts, representative of trade unions, psychologists, academics, psychosocial risk experts, and employers' representatives.

The stakeholders participating in the local consultation groups expressed their views on different emergent topics, including the project in general, the strengths and weaknesses of the intervention materials, the design, lay-out and usability of the intervention and the use of potentially problematic terms or concepts. They were also asked specifically to list some aspects they felt could be improved or should be changed or added in the final version of the intervention. The consultations generated many relevant remarks and questions that are useful to consider when developing the digital intervention. The overall impression of the project, its aims and the intervention material were very positive. The consulted stakeholders liked the idea to raise awareness about mental health and they were enthusiastic about the focus on the workplace. In this context, the stakeholders pointed out that the actual involvement of the employers/organizations should be strengthened. The stakeholders also liked the approach of a mobile phone application with interactive elements as a tool for promoting and monitoring the mental health of individuals, although they also expressed some concerns about the use of an application. The stakeholders stressed the importance of developing an attractive and user friendly lay-out for the intervention. Each of these concerns was acted upon, to improve the development and the implementation of the intervention.

Finalised target-user profiles, so called personas, included three fictional characters representing specific user-groups based on information from secondary sources (e.g., official government and organisational records, studies) about mental health and digital literacies, socio-economic status, occupation, educational background, goals, motivations, interests, and common life challenges. From the profiles created, we extracted three main user personas to focus on. We used key aspects such as level of digital literacy and level of mental health awareness, and demographic and work backgrounds to differentiate between our personas. The final result of the process was a beta version of the prototype which was ready for user testing leading to iterations and improvements in design before the final release for evaluation in a planned RCT (Olaya et al., 2021).

### 4.5 Usability testing

The prototype was tested by ten participants in the United Kingdom, 40 % were 35–44 years of age, 20 % were 25–34, 20 % were less than 24, 10 % were 55–64, and 10 % were more than 65. 70 % were female, and 80 % had a bachelor's degree or above education. The majority of survey responses expressed favourability toward the apps appearance and critical feedback mostly surrounded limitations of the prototype design (e.g. that it is not very navigable and has minimal material).

**Fig. 9:**
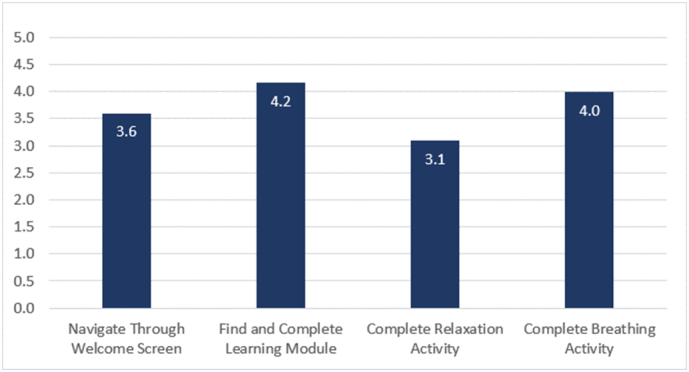
Ease of use ratings by task.

The beta version was tested by 31 participants from the four countries. The majority were highly educated, females, mostly aged between 35 and 45. There were 4 testing tasks along with follow up questions regarding ease of use, visual presentation, and participant suggestions. Task 1 was navigating through the welcome screens at the beginning of the app; task 2 was a learning module that participants needed to find and complete; task 3 was a relaxation activity; and task 4 was a breathing activity. 30 (97 %) completed task 1; 31 (100 %) of participants completed task 2; 24 (77 %) completed task 3; and (93.5 %) completed task 4. Ease of use ratings are summarised in Fig. 9.

In addition to the tasks and ratings for ease-of-use, participants were asked for their suggestions, which were analysed. A list of conclusions regarding positive feedback and constructive feedback was compiled and some parts of the testing process and development were adapted. The triage and algorithm described above were also tested to check if users of the app were guided toward the appropriate parts of the app as intended based upon their answers in the triage. In general, flow through the app was reported to be simple and users found the app relevant and easy to use. Participant suggestions also led to further changes to the app navigation, such as the addition of a “finish” button, and to ease of use, such as increasing the font size of the text. While there were constructive recommendations and feedback, overall, the results show a favourability for the look, navigation, and material in the app. Usability testing participants especially appreciated the interactive features and said they could see themselves using or recommending the app. The final version of the intervention is available in a desktop version and in a mobile app. More information about the qualitative testing is provided in the supplementary file.

## 5 Discussion

This study was a description of the design and development of the EMPOWER digital intervention prototype and beta version. Usability testing results showed that users were able to complete tasks in the app and that they liked the design.

As mentioned in the rationale, while there are a few digital interventions already on the market, they typically target individual issues, such as depression alone, rather than focusing on comorbidities and integrative approaches that address mental health symptoms and work participation after illness (Stratton et al., 2021; Heber et al., 2017; Deloitte Global Human Capital Trends, 2017). The triage design for the EMPOWER app targets symptomology of individual users and is based upon validated risk assessment questionnaires. The resulting protocol allows for variations in support level and material offered depending on participant need as well as specific modules to address comorbidity. This design provides an innovative approach to addressing workplace mental health concerns and targeted support of employees as well as managers and employers for work related problems, unlike most digital interventions on the market currently that focus on employees only (Stratton et al., 2021; Heber et al., 2017; Deloitte Global Human Capital Trends, 2017).

The focus during development on gender and cultural differences that could impact successful implementation, while also translating the vocabulary and grammar of the text, is a unique feature of the EMPOWER intervention. The intervention was carefully adapted to ensure usability, acceptability, and adherence for each country as well as comparability of the material across all four settings. Another unique feature was the participation of local consultation groups to provide feedback on the project in general, strengths and weaknesses of the intervention materials, design, layout, usability, and any use of potentially problematic terminology or concepts. Changes made following usability testing will potentially further enhance the usability and effectiveness of the app.

### 5.1 Limitations and strengths

The EMPOWER digital intervention is subject to limitations inherent in any digital intervention. It requires the use of a computer and smartphone, as well as internet connectivity, digital literacy, and general literacy, to access all aspects. While the app was designed to be personalised to individual user needs, it was not designed to address suicidal ideation or intent. Users who identify as experiencing suicidal ideation are allowed to use the app but are encouraged to speak with a medical professional and advised the app is not designed to address their mental health needs. As with any digital intervention of this kind, it is impossible to meet the needs of all users, although the EMPOWER digital intervention is designed to meet the needs of employees from a variety of contexts.

Additionally, the usability testing did not provide users with the full app but core parts of it in 3 iterations. The testing was done qualitatively in a rather small and homogenous group, with the majority being female employees with a higher education background. This may not be representative of the end-user population. These limitations are appropriate for a test of this kind but should be considered when examining the results of the RCT that is planned to follow with this app. We also did not seek feedback from employers in the usability testing or explore how employees would feel about grouped data on the psychosocial context being shared with employers.

It will be a limitation of the future RCT that providing employers with feedback on employees might pose ethical issues. We will only provide anonymous and aggregated feedback regarding psychosocial risks at a company level together with tailored recommendations to reduce these risks. This will only be provided if 10 or more employees have provided information about the psychosocial context at the workplace. The personal information as to levels of anxiety, depression etc. will in no way be shared with employers. Employees will be informed about this before signing the informed consent, and as participation will be completely voluntary, this might cause stress for the employees and limit their participation, which might result in participation bias in the future trial.

Despite these limitations, designing a digital intervention to address work stress and psychological symptoms that contribute to presenteeism and absenteeism while improving wellbeing for employers and employees, is highly innovative and timely. Its multi-module and triage – algorithm led design provides a highly individualised and unique user experience. While the ongoing pandemic presented a challenge to the design process, it also increased the need for support at the workplace for workers and companies, as COVID-19 and home confinement has had a negative impact on mental health while also increasing use of digital devices (Ammar et al., 2021). The EMPOWER digital intervention's development is well-timed to provide a much-needed intervention that is highly accessible to a high percentage of the working population. Our documentation and publication of the design and development process, along with our use of a cultural validation approach in co-design with stakeholders and end-users strengthen the resulting intervention. Also, the usability testing before the planned start of the evaluation in an RCT is a unique feature of the EMPOWER project and app development.

### 5.2 Conclusion

The EMPOWER digital intervention is a tailored multimodal intervention addressing wellbeing, work stress, mental and physical health problems, and work productivity. This will be used in a planned RCT to evaluate its effectiveness.

## Ethical approval

The participation of stakeholders in the usability testing and cultural adaptation have been approved by the ethics committees of Fundació Sant Joan de Déu (PIC-39-20), Turku University Hospital (PIC-993966082), University of York (HSRGC250321) and Institute of Occupational Medicine, University of Lodz (9/2020). All participants for the translation and cultural validation gave their informed consent to participate in the online focus groups and agreed to be videotaped.

## CRediT authorship contribution statement

The manuscript text was written by: CvdF-C, JS, BO, JG, KvA, CV, OBC, LGR, RMB, DM-K, MS, PN, LH-vR, FvK, and MdM. Review and editing of the manuscript text was completed by all authors. All authors approved the final manuscript. Funding acquisition for the EMPOWER project was conducted by: BO, CV, CM-K, MS, JMH, LH-vR, and CvdF-C. Methodology for the manuscript was created by: JS, BO, JG, KvA, CV, OBC, LGR, RMB, EV, DM-K, MS, PN, LH-vR, FvK, MdM, JMH, and CvdF-C. Conceptualisation of the manuscript and article was completed by: JS, JG, KvA, CV, OBC, LGR, RMB, DM-K, MS, PN, LH-vR, FvK, MdM, and CvdF-C. Figures were prepared by: JS, JG, OBC, EV, and DM-K. All authors approved the final manuscript.

## Funding

This project has received funding from the 10.13039/501100000780European Union's Horizon 2020 research (https://ec.europa.eu/programmes/horizon2020/en/home) and innovation program under grant agreement No 848180, and the 10.13039/501100000925National Health and Medical Research Council (NHMRC) of Australia under Grant Agreement APP1195937.

Beatriz Olaya is supported by the Miguel Servet (CP20/00040) contract, funded by the 10.13039/501100004587Instituto de Salud Carlos III and co-funded by the 10.13039/501100000780European Union (ERDF/ESF, “Investing in your future”). Mireia Félez Nóbrega is supported by the 10.13039/501100004587Instituto de Salud Carlos III, (postdoctoral grant CD20/00036). Carlota de Miquel has received funding in form of a pre-doctoral grant from the 10.13039/501100002809Generalitat de Catalunya (PIF-Salut grant, code SLT017/20/000138).

The funders played no role in study design, data collection and analysis, decision to publish, and preparation of the manuscript.

## Declaration of competing interest

Christina van der Feltz-Cornelis received honoraries from the Lloyds Register Foundation and Janssen UK. Oriol Borrega Cepa and Laura González Recio have a paid consultancy and development of the Spanish based project “SOM360 - Salud Mental” (https://www.som360.org), participation in the European Project “Share4Rare” as analyst and developer (https://www.share4rare.org), and participation in the European Project “ACADOM” as analyst and language expert (https://www.omada.es/blapp/). All other authors have no conflicts of interest to report.

## Appendix A Supplementary data

Supplementary material Image 1
