# Supplementary material for: Design and development of a digital intervention for workplace stress and mental health (EMPOWER)
Source: Internet Interv. 2023 Nov 4;34:100689. doi: 10.1016/j.invent.2023.100689 (PMC10694565; doi:10.1016/j.invent.2023.100689)
Supplement: Supplementary file 1 — Supplementary material [file mmc1.pdf]

Supplementary material – Outcomes of the user-centred design process and testing

Van der Feltz-Cornelis et al. Internet Interventions 2023

Phase I – Design of the intervention

Content development

1. A component of mental health promotion at the workplace that includes raising awareness about workplace mental health
2. A component to screen for psychosocial working conditions and provide recommendations to address those for employers and employees
3. A component to screen for mental health, physical health, and absenteeism of employees and provide recommendations to promote well-being and mental health in employees
4. A work functioning component to help deal with work-related problems such as presenteeism and frequent absenteeism
5. Anti-stigma and raising awareness website

Translation and cultural adaptation – forward translation & negotiated consensus

Following a cultural adaptation protocol developed for this purpose, the content of the intervention was translated to the native language of the RCT sides (Polish, Finnish, Spanish) with cultural adaptation to make content more understandable for the participants from these settings. Subsequently, negotiated consensus took place, meaning that there was a discussion between research teams on challenging concepts and documentation of such modifications to maintain the core parts of the materials as well as allow for cultural sensitivity.

Technical development

Basic user-experience requirements were determined based on extensive analyses including assumptions about users, results from published literature on mental health apps, and past reports of user experience with comparable apps.

Subsequently, several persona-based profiles were created that differed in terms of needs, goals and context of usage. Finally, we used key aspects such as level of digital literacy and level of mental health awareness, and demographic and work backgrounds to differentiate between our personas.

These profiles were designed to represent the needs of prototypical target users of the app and helped us build a beta version of the digital intervention, which we tested during the usability testing.

Phase II – Stakeholder consultations, expert evaluation and prototype pre-testing with potential end-users

Content development - Stakeholder consultations

A total of 25 stakeholders participated in the local consultation groups, representing the following fields: occupational health experts, representative of trade unions, psychologists, academics, psychosocial risk experts, and employers' representatives. A detailed overview of the stakeholder profiles per country can be found in Table 1.

Table 1 – Key stakeholder profiles involved in stakeholder consultations

Poland		Finland		Spain	
M	Occupational physician	F	Occupational health expert	M	Professor Public Health
F	Psychologist, Academic expert	F	Occupational physician	M	Psychologist of insurance company
M	Patient organisation	M	Senior Adviser, Working Life	F	Manager of Department of Occupational Risk Prevention
M	Vice president Business Centre	M	Academic, Professor	M	Representative of Trade Union
M	Plant manager	F	Consultative officer	M	Psychologist of insurance company
F	Representative of Trade Union	M	Representative Finnish Partnership for Mental Health	F	Manager HR department
F	Occupational Health and Safety specialist	F	Psychosocial factor expert	F	Director of Digital Health Service of insurance company
F	Solicitor, legal counsel	F	Rehabilitation planner		
F	Coach Psychology for Business				

The following questions were discussed in the stakeholder consultations:

1. What is your general impression of the EMPOWER project?

2. What do you like most about the intervention materials (list 3 things)?

3. What do you like least about the intervention materials (list 3 things)?

4. What is your opinion on the design and the layout of the intervention?

5. Do you think that the intervention is easy to use?

6. Did you come across any content/words/expressions that are unclear?

7. What are your thoughts on how the intervention could be improved?

8. If you could make one change to the intervention, what would it be?

9. Did you notice any obstacles that would prevent an individual to use this intervention?

10. Do you feel like anything is missing from the intervention materials and/or are you aware of any other materials that could be included?

Table 2 – Findings stakeholder consultations

Stakeholder feedback	Improvement / Action undertaken
1. The stakeholders stressed the fact that companies should be more actively involved in the procedure and should receive input on how to implement the intervention and how to follow up on the recommendations that are provided.	- This is why we provide the employers with information on how to implement the EMPOWER intervention and the different steps that follow afterwards (a participative stepwise approach).<br>- We also include references to country-specific institutions or consultants that could help the companies with this.
2. Some stakeholders advised to add information which allows employers to put the recommendations into perspective. What if only a small number of employees from a company participate? This may be enough for a very small company to generate recommendations, but not for medium-sized or large companies.	- The implementation of a message for employers considering the share of participating employees in the company, if information about the size of the company is available.
3. Some stakeholders were of the opinion that the intervention was focussed too much on mental health problems and disorders and that more attention should be paid to the actual promotion of mental health and well-being (positive framing).	- This concern can be explained by the fact that only fragments of the content were presented to the stakeholders, which gave them the (wrong) impression that the app focuses only on symptoms and disorders such as depression and anxiety. In fact, the content of the complete EMPOWER intervention has a good balance between negative and positive aspects of mental health.
4. Another suggestion related to content that was made by some stakeholders is to include more detailed and specific information in the app.	- Of course, it is also crucial to not provide too much information within the app, to avoid that people are discouraged by the volume of the content. This is why we include links in the app for those people who want to learn more about certain topics. In that case, people who are interested can click on the link and others can just continue with the app.

Translation and cultural adaptation – pre-testing & modification

Three potential users (employees), one employer representative, and one legal expert from each country-setting reviewed the translations and provided feedback that informed any updates to the translated text.

The considered adaptations made included ways of rephrasing parts of the source materials or adding definitions and synonyms to difficult concepts to ensure comprehension among the target population.

Other factors of usability, like the structure of the text, which was deemed overly schematic by potential end-users, and overall framing, which end users felt was too ‘negative’ (i.e., focus on ‘illness’ rather than ‘wellness’) were discussed and adapted.

Unique practices and ‘social codes of conduct’ within each setting were also taken into account (e.g., in the Spanish text, the mention of standing desks was limited due to it not being a common practice in Spain).

Further adaptations were related to the regulations within the field of occupational health and safety for each of the three settings (e.g., the equality policy and occupational health care were elaborated on in Finnish documents, and specific employee break regulations were clarified in Polish documents).

Technical development

Several high-fidelity functional prototypes were designed and developed as a non-interactive web-based prototype in English. This prototype version underwent usability testing by ten potential end-users from the United Kingdom (see Table 3).

Table 3 – Participants prototype testing

	**United Kingdom**
Gender	
Male	30%
Female	70%
Age	
≤ 24 years	20%
25-34 years	20%
35-44 years	40%
45-54 years	
55-64 years	10%
≥ 65 years	10%
Education level	
University	80%
Others	20%

The majority of survey responses expressed favourability toward the apps appearance and critical feedback mostly surrounded limitations of the prototype design (e.g. that it is not very navigable and has minimal material).

Based on the prototype testing the following conclusions were drawn:

Positive:

- Most of the tasks have been completed successfully
- The participants overall understand the information structure and where to go to do an action or find some content.
- Bad feedback is more related to static content and incompatibilities with the browser than the usability (inherent to way of presenting at this stage)
- People will use it if the content is personalized enough as we aim
- The colour scheme is liked.
- The participants would recommend the app!

Table 4 – Results of prototype testing (Usability and Branding)

Task	Difficulty according to participant	Recommendation for improvement & Action undertaken
Usability		
1. It is believed that regularly tracking your mood helps you understand your emotions better, detect emotional patterns and improve your mental health. How are you feeling today? Track it in our app!	2.6/5 (easy to average)	Only half of participants understood that after completing the task, you unlock new content about your activity and health –> Notifications and more context associated to what the user answered that day will help.
2. The app encourages you to practice healthy habits and track them to achieve it. We offer pre-established habits for you to choose, such as work, lifestyle, sleep, or you can also write your own. Would you start working on a pre-established habit about LIFESTYLE?	2.8/5 (average)	- Some users the + button was not displayed, so they could not do the task<br>- Maybe, the word pre-established was not understood<br>- Preference for more personalization instead of predetermined options like the "Habits"<br>–> Giving more context about Empower and what we need the participants to do in the testing.
3.1 As you may already know, the study lasts 7 weeks. This is the amount of time you will be using the app and we will track your progression. Which week of the intervention does the prototype say you are at currently? Find it out!	2.75/5 (easy to average)	–> Welcoming every week as it starts to remember users this is a 7-week path and contextualize them how much they did.
3.2 To motivate end-users to fulfil the 7-week intervention, they are rewarded with medals for their effort and consistency. Find the medals you've received!	4/5 (difficult)	- In some browsers, medals were not displayed<br>- Since they did not win those previous medals (so it was not anything personal), maybe they did not think they would have it in their Profile section<br>–> In the real use of the app, users will receive a notification when they get a medal and this will direct them to the profile, so they will learn where to find the medals.
4.1 There is a tool in Empower called Problem solver where you can detect a problem, set a goal and track your progress in meeting the goal. Find this section and start a new problem to set your goal!	2/5 (easy)	–> Suggesting users to do the Return to work capsule as a guidance for the first time they use the tool, as it is rather complex with several steps.
4.2 "The app also offers knowledge capsules to learn about mental health topics. Now, let's learn about anxiety!	3/5 (average)	–> Improving the intro page of each capsule by adding: time, benefits, who's it for and an index.

Branding		
What suggestions would you recommend to ensure the branding gives a more positive impression?	3/5 (positive look & feel)	–> Initially, purple was used for interaction and orange for static elements. It is however more intuitive to use illustration tones for type of activity, for instance, orange is learning and blue are habits.

To improve in usability:

- Better correlation between the tasks and its feedback in Today section.
- Two screens more in Onboarding for specific features or sections. People seem confused about what, how to do and why when they land the app.
- Limiting the content shown in Today section so that it does not confuse the user about its aim.
- 'Welcome to the week x!' message at the beginning of every week
- Percentage of completion as a remember of the intervention as a 7-week path
- Notification when a medal is won

Lessons learned about the testing:

- It is important for the users that the content is oriented to their profile to take full advantage.
- Users need more context of what Empower is and what we need from them in the testing
- The testing pretends the participant are in the middle of the intervention (when they are fully adapted to the app) while the truth is that this is the first time they use the app and there is no learning curve.
- We should have put more emphasis on explaining to the participants that this testing was static.

Phase III – Testing beta version and user journey

Technical development

Qualitative usability testing was performed on a software-based interactive beta version of the EMPOWER app. The beta version was tested by 31 participants from Spain, United Kingdom, Poland and Finland. The majority were highly educated, females, mostly aged between 35 and 45.

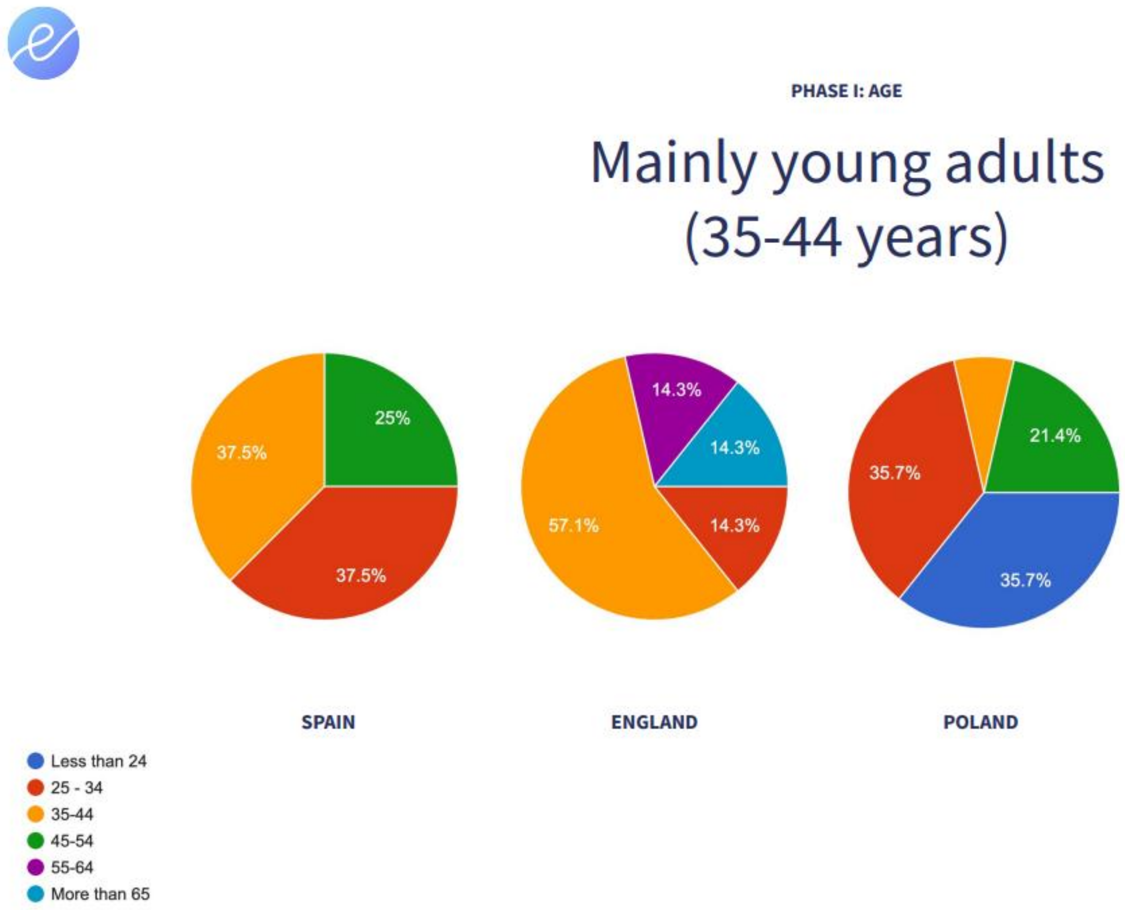

For details, see Table 5. Participants beta version and user journey testing

	**Spain**	**United Kingdom**	**Poland**
Gender			
Male	37,5%	28,6%	50%
Female	62.5%	71,4%	50%
Age			
≤ 24 years			35,7%
25-34 years	37,5%	14,3%	35,7%
35-44 years	37,5%	57,1%	7,2%
45-54 years	25,0%		21,4%
55-64 years		14,3%	
≥ 65 years		14,3%	
Education level			
Primary school			
Secondary school			
NVQ or equivalent	25%	14,3%	50%
University	62,5%	85,7%	50%
Others	12,5%		

Table 6 – Outcomes beta version and user journey testing

Task	Difficulty	Recommendations for improvement / Actions undertaken
Usability		
1. The app first guides you through 3 screens that introduces you to the EMPOWER app. Please complete this introduction without selecting Skip in the top-right corner of the screen	3.6/5 (easy to average)	n/a
2. There is a learning capsule That teaches us how to cope at work while experiencing mental health problems. Please find the work learning capsule and complete it	4.16/5 (easy)<br>- Some users found it difficult to exit the module to the main page.<br>- Going back took a user back to the beginning of the session.<br>- When users accidently give a wrong answer, it seems impossible to correct.<br>- One user was not sure if the questionnaire had to be "answered" or, that just clicking on the different options was enough.<br>- A Finish button is missing at the end of the capsule.<br>- In the end, it would be good to have a button that takes us back to the beginning.	- Specifying the back action with ‘Back to Learn’<br>-Not using the back arrow for “previous” and “next”.<br>-Confirmation by first selecting the option and then clicking “Answer” in all kind of questionnaires to align the user’s behaviour<br>-Adding a finish button at the end of the capsule which replaces the ‘Next’ button and adding ‘Back to’ to the upper navigation.
3. The app features a relaxation exercise that could be great for those moments when you need to find some calm. Please find this exercise and try it for 5 minutes.	3.1/5 (average)<br>- The relaxation exercise was said to be 5 minutes long but the recording was 10 minutes long	- Adapting all the call to actions to the true length of the audios.
4. The app also features a breathing exercise that would also be great for those moments when you need to find some calm. Please find this exercise and try it for 5 minutes	4/5 (easy)<br>- Maybe there could be an audio for the Exhale and Inhale, because many times the exercise is done without looking at the phone<br>- At first the graphic matched the words, but then the words started changing and were out of sink with the circle.	- Audio guidance has been implemented<br>- Technical issue with out-of-sync audio was fixed.

Table 7 – End-user feedback based on predetermined statements

Statement	Strongly disagree	Disagree	Neutral	Agree	Strongly agree
I think that I would like to use this app frequently	9,7%	19,4%	29,0%	32,3%	9,7%
I found the app unnecessarily complex	35,5%	45,2%	9,7%	9,7%	-
I thought the app was easy to use	6,5%	9,7%	16,1%	35,5%	32,3%
I think that I would need the support of a technical person to be able to use this app	61,3%	32,3%	6,5%	-	-
I found the various functions in the app were well integrated	3,4%	13,3%	16,7%	53,3%	13,3%
I thought there was too much inconsistency in this app	35,5%	45,2%	16,1%	3,2%	-
I would imagine that most people would learn to use this app very quickly	3,2%	3,2%	16,1%	32,3%	45,2%
I found the app very cumbersome to use	50,0%	26,7%	20,0%	3,3%	-
I felt very confident using the app	6,5%	6,5%	19,4%	41,9%	25,8%
I needed to learn a lot of things before I could get going with the app	61,3%	29,0%	6,5%	3,2%	-

Based on the prototype testing the following conclusions were drawn:

Positive:

- All tasks were successfully passed!
- The participants understand how the app works and navigate correctly
- The practice part is the favourite one, meaning that users prefer the more interactive material
- Testers like the illustrations and find all the look & feel warm

To improve in usability:

- In capsules: Increasing the size of the text; Reviewing the capsule navigation; Improving the call to action to go back; Answer confirmation for all the questionnaires; Adding a Finish button at the end of the capsule
- In tools: Adding music to the breathing exercise; Fixing some technological issues; Aligning the time of the audios with their call to actions; Considering in the future to present the content with audio

Lessons learnt about testing:

- The context about the testing really helped the participants this time to understand what we needed from them, it avoided confusion and starting in disadvantage

Phase IV – Final adaptations

Technical development

Based on excessive further testing by the EMPOWER consortium, the following issues were discovered and resolved:

- Mistakes in the personalization algorithm; some users did not see the contents they were expecting to their profiling
- Two content capsules were not uploaded to the production version of the app
- The widget for inputting dates is cumbersome
- Fallback solutions for edge cases were missing. For instance, if a user runs out of proposed contents in their TODAY screen
- Some untranslated strings and textual mistakes in different languages
- Some users were not getting the invitation emails

Summary of testing

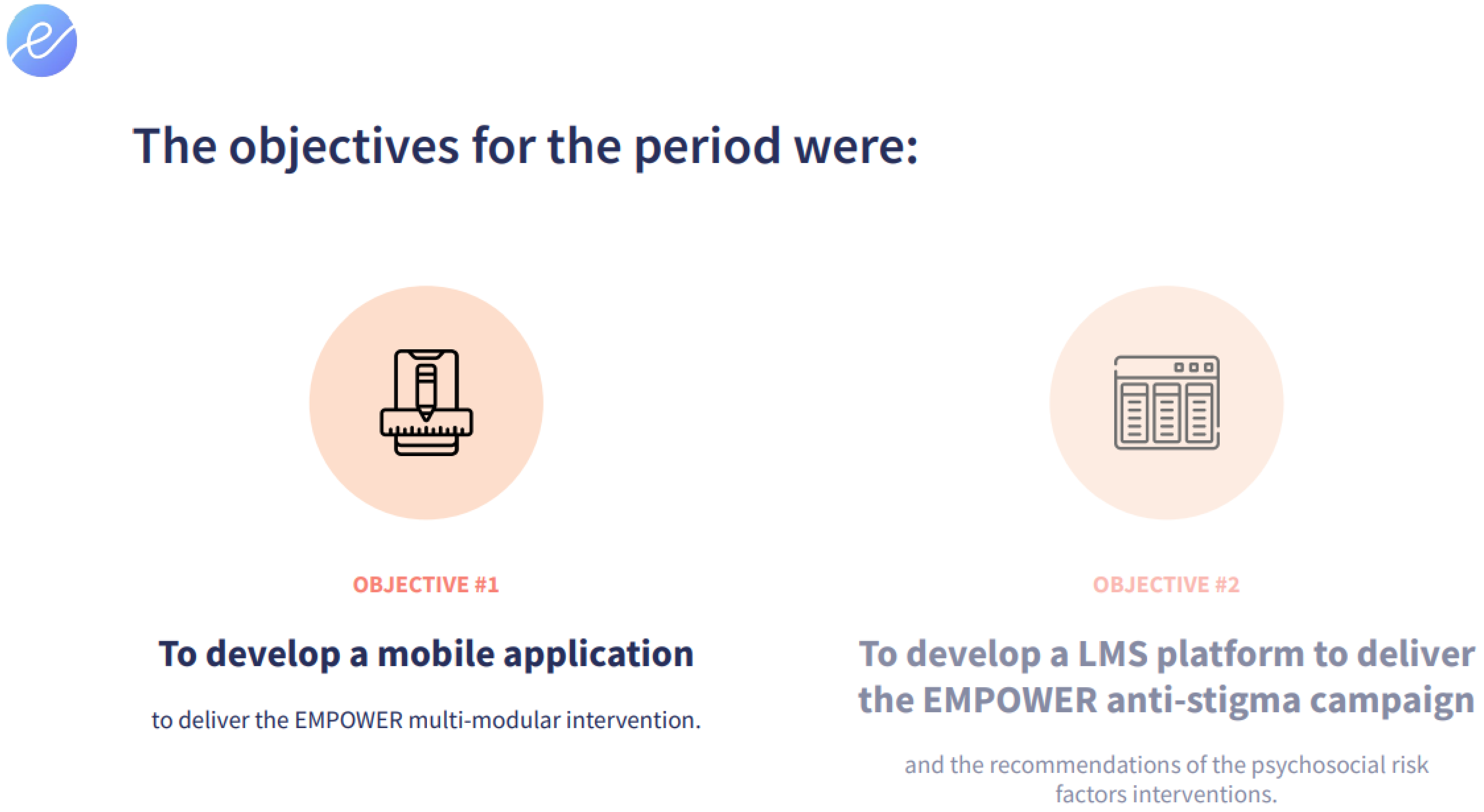

1. Objectives

OBJECTIVE #3

To test the usability and acceptability

of the EMPOWER multi-modular intervention in a pilot study.

OBJECTIVE #4

To conduct a series of focus groups sessions with employers, employees and stakeholders advisory board

To test and play the first EMPOWER app prototype. Future finements of the modular eHealth platform will be conducted according to participants' inputs.

OBJECTIVE #3

To test the usability and accessibility of the EMPOWER platform

Phase I: External testing with users

What was detected?

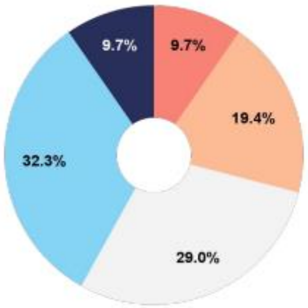

"I think that I would like to use this app **frequently**."

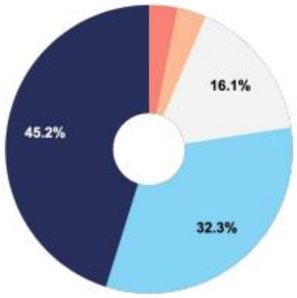

"I would imagine that most people would **learn to use** this app very quickly."

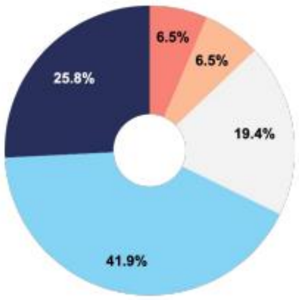

"I felt very **confident** using the app"

Strongly disagree · Disagree · Neutral · Agree · Strongly agree

OBJECTIVE #3

To test the usability and accessibility of the EMPOWER platform

Phase I: External testing with users
What was detected?

All the tasks were successfully passed!

Both in testing I and testing II

The participants understood the information structure, how the app works and navigate correctly

The practice part is the favorite one, so users prefer to see more interactive material

OBJECTIVE #3

To test the usability and accessibility of the EMPOWER platform

Phase I: External testing with users

What was detected?

In capsules, we needed confirmation for all the questionnaires

In tools, we received the suggestion of adding music to the breathing exercise

Which we finally implemented!

Some technical issues had to be fixed

OBJECTIVE #3

To test the usability and accessibility of the EMPOWER platform

Phase II: Internal testing with EMPOWER members
What's been detected?

Mistakes in the personalization algorithm

Where some users did not see the contents they were expecting according to their profiling

Two content capsules were not uploaded to the production version of the webapp

The widget for inputting dates is cumbersome

OBJECTIVE #3

To test the usability and accessibility of the EMPOWER platform

Phase II: Internal testing with EMPOWER members
What's been detected?

Fallback solutions for edge cases were missing

Example: If a user runs out of proposed contents in their TODAY screen

Some untranslated strings and textual mistakes in different languages

Some users were not getting their emails

OBJECTIVE #1

To develop a mobile application and LMS platform

What's been changed?

The following changes respond to new requirements or situations which arise during the development of the webapp.

1. Adaptation of the Assessment tool to meet the requirements of the EQ-5D-5L surveys by EuroQOL

The changes adopted have been implemented across the whole tool, not only to this particular survey.

2. Alternative system for sending invitation codes to participants in the UK, and specific version of the IC for UK participants

To meet the new set of legal requirements that stem from the inclusion of companies in the UK, alternative strategies for handling user data at specific points have been implemented. Invitation codes for the UK are generated using dummy emails and sent to participants *offline* (not using the platform's backend).

OBJECTIVE #1

To develop a mobile application and LMS platform

What's been done?

1. Profile section

Implementing a section that allows participants to change preferences and see relevant information, and to implement a gamification system to increase engagement with intervention

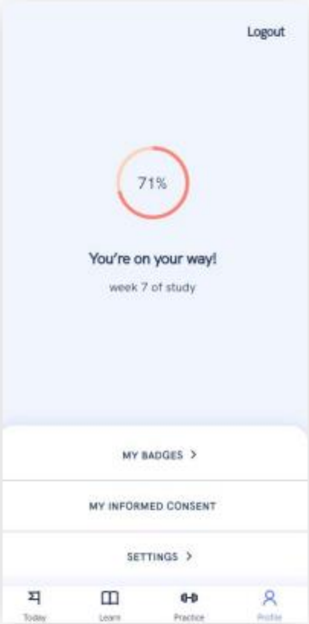

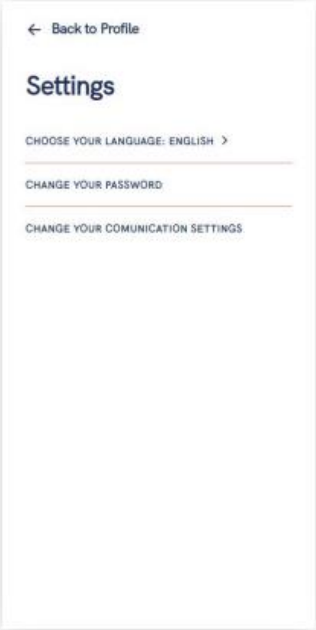

OBJECTIVE #1

To develop a mobile application and LMS platform

What's been done?

The names of the badges will be changed after the feedback from consortium members received during the last round of testing.

2. Badges

3-level-rewards to the users for completing tasks by using the tools in the app and reading the content chapters (located in profile).

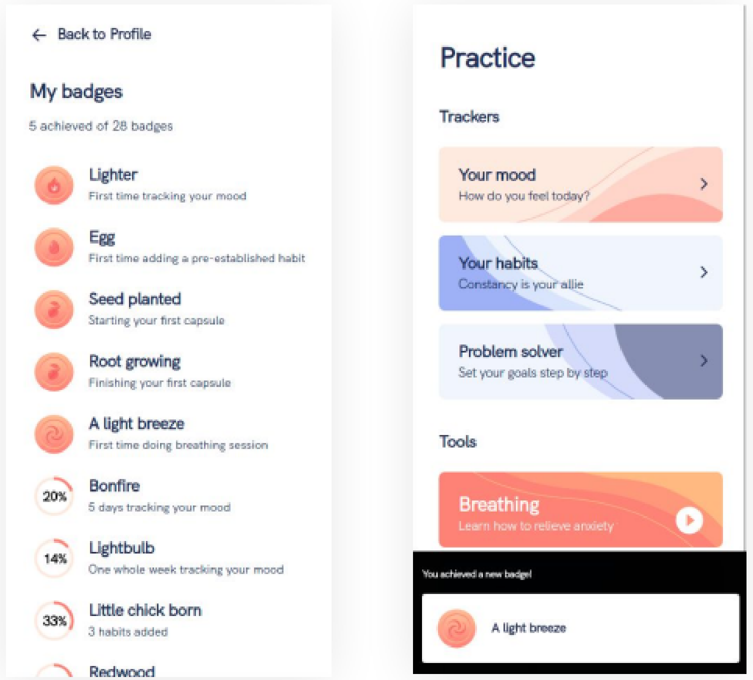

OBJECTIVE #1

To develop a mobile application and LMS platform

What's been done?

The basic algorithm has been changed too in order to correct malfunctions and to increase robustness and performance.

3. Algorithm to tailor PRS content to users according to their profile

To deal with Psychosocial Risk Scale survey and the recommendations stemming from it, both for the participants (employees) and for the companies (employers).

The feedback to employers is located in a specific access point in the company backend.
